# Adsorption of phenol and bisphenol-A by activated carbon and graphene oxide: a comparative study

**DOI:** 10.1007/s11356-025-37204-7

**Published:** 2025-11-26

**Authors:** Amina BiBi, Sami Sayadi, Mohammed Abu-Dieyeh, Nabil Zouari, Mohammad A. Al-Ghouti

**Affiliations:** 1 Department of Biological and Environmental Sciences, College of Arts and Sciences, Qatar University, Doha, Qatar; 2 Center For Sustainable Development, College of Arts and Sciences, Qatar University, Doha, Qatar

**Keywords:** Phenolic compounds, Adsorption, Activated carbon, Graphene oxide

## Abstract

The adsorption of phenol and bisphenol-A (BPA), two prominent environmental pollutants, by two distinct adsorbents—activated carbon (AC) and graphene oxide (GO)—was investigated through a comparative study. Batch adsorption experiments were conducted to evaluate and compare the adsorption capacities of AC and GO for phenol and bisphenol-A removal from water. The adsorbent characterization was done using various physicochemical studies, including FTIR, SEM, TEM, RAMAN spectroscopy, and BET surface area analysis. The effects of various experimental parameters, including pH, initial pollutant concentration, temperature, and ionic strength, were examined to explain the adsorption mechanisms and optimize conditions for efficient phenol removal. The results showed that both AC and GO exhibited adsorption capacities for phenol and bisphenol-A removal, with AC showing more resilience at various experimental conditions. The maximum adsorption capacities reached approximately 39.37 mg/g for phenol and 172.41 mg/g for BPA on AC. At the same time, GO achieved around 7.69 mg/g for phenol and 70.92 mg/g for BPA under optimal conditions. Furthermore, the comparative study provided insights into the advantages of AC and limitations of GO as adsorbents for phenol and bisphenol-A removal, highlighting their potential applications in wastewater treatment and environmental remediation efforts.

## Introduction

Water contamination by organic pollutants has emerged as a critical environmental challenge over the last few years, driven by rapid industrialization, urbanization, and inadequate waste management practices (Babuji et al. 2023; Wen et al. 2017). Among these pollutants, phenolic compounds, including phenol and bisphenol-A (BPA), are of particular concern owing to their widespread occurrence, persistence, and adverse effects on ecosystems and human health. Phenol, a simple aromatic compound, is generated by various industries such as petrochemicals and pharmaceuticals. In contrast, BPA, a synthetic compound used in plastics and epoxy resins, is released into the environment from domestic and industrial sources. Both compounds are regularly detected in surface water, groundwater, and wastewater, posing significant risks to aquatic life and human populations (Anku et al. 2017; Bibi et al. 2023; Loganathan et al. 2023).

**Fig. 1:**
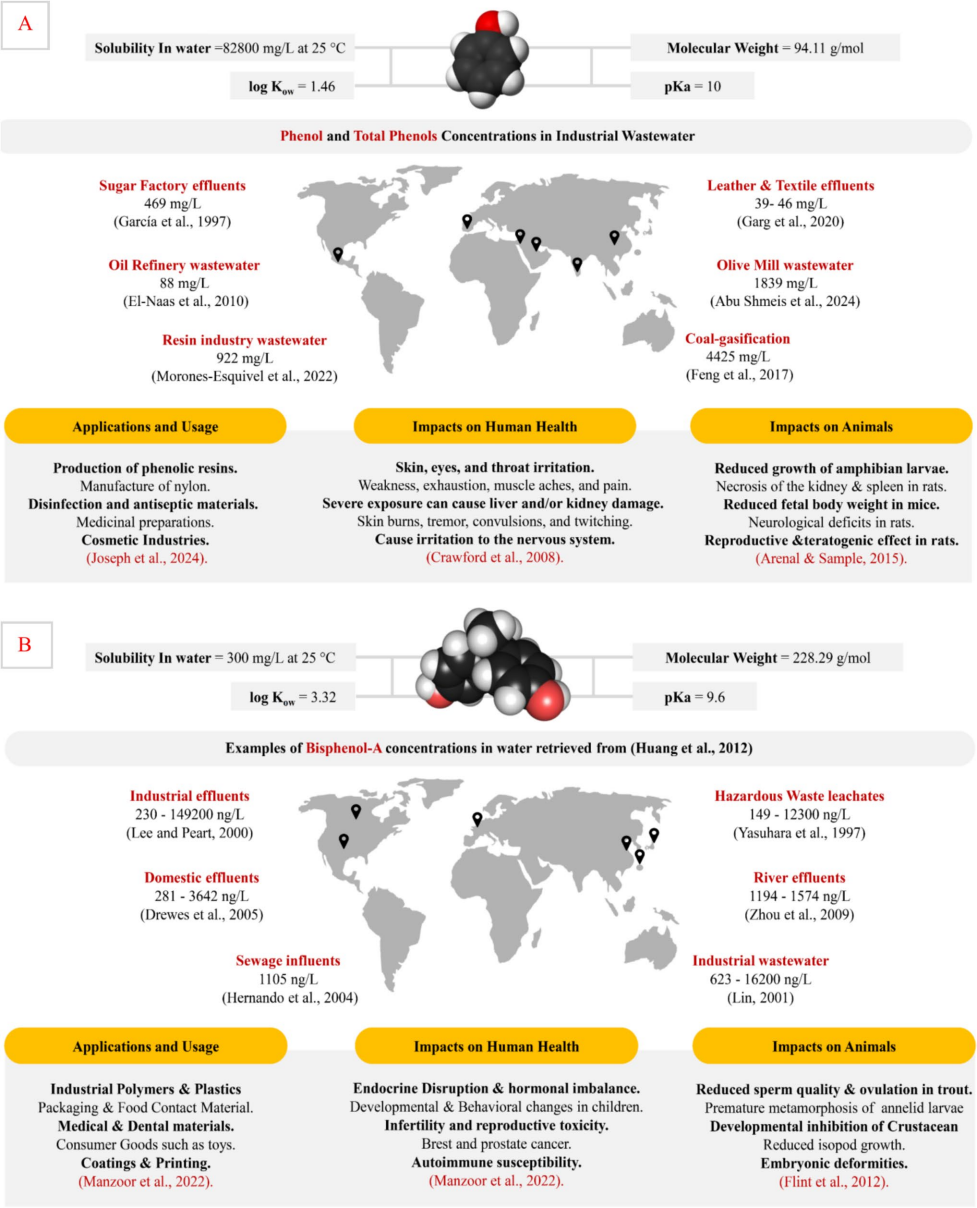
Physicochemical properties, industrial relevance, environmental occurrence, and toxicological impacts of phenol and bisphenol-A across global regions

Phenol and BPA represent two classes of environmental pollutants. Phenol is extensively used in the manufacturing of resins, antiseptics, pharmaceuticals, and cosmetics, contributing to its frequent availability in industrial effluents. As illustrated in Fig. 1(A), phenol concentrations in wastewater can vary significantly by source and region (Feng et al. 2017; Garg et al. 2015). Due to its high-water solubility (82,800 mg/L) and moderate hydrophobicity (log K_OW_ = 1.46), phenol remains mobile in aquatic systems, raising environmental and health concerns. Unfortunately, exposure to phenol has been linked to dermal and respiratory irritation, organ toxicity, and neurotoxic effects in humans, as well as growth and reproductive impairments in animals (Arenal and Sample 2015; Crawford et al. 2008). The United States Environmental Protection Agency (EPA) classifies phenol as a priority pollutant, with a recommended limit of 1 µg/L in drinking water (Saputera et al. 2021). BPA, on the other hand, is a synthetic compound primarily used in the manufacturing of epoxy resins and polycarbonate plastics (Hahladakis et al. 2023), found in products ranging from food containers (Khalili Sadrabad et al. 2023) to medical devices (Guimarães et al. 2023). Its release into the environment occurs through leaching from domestic products, industrial discharges, and landfill leachates. As summarized in Fig. 1B, BPA is known for its endocrine-disrupting properties, which can interfere with hormonal systems in humans, leading to reproductive, developmental, and metabolic disorders (Ramakrishna et al. 2021). Figure 1B also shows that BPA has been globally detected in water bodies at concentrations typically ranging from nanograms to micrograms per liter (Huang et al. 2012), highlighting the urgent need for effective removal strategies, especially considering its maximum allowable dose level of 157 µg/day (Goodman et al. 2017; Tarafdar et al. 2022). Given their physicochemical characteristics and toxicity, both phenol and BPA require targeted interventions to mitigate their environmental and health impacts.

The need for practical, sustainable, and cost-efficient technologies to eliminate these contaminants from water has thus become a priority in recent years. The treatment of phenol and BPA-contaminated water has been approached through various methods, including advanced oxidation processes (AOPs), biological degradation, membrane filtration, and adsorption (Bibi et al. 2023). AOPs, such as electrooxidation, can degrade these pollutants into less harmful byproducts but require high energy inputs and may generate secondary pollutants (Ma et al. 2024). Membrane filtration, while efficient, is limited by high operational costs (Ramos et al. 2021). Adsorption, in contrast, offers a balance of efficiency, simplicity, and adaptability, making it one of the most commonly adopted practices for organic pollutant removal (Magdy et al. 2021). The efficiency of adsorption relies significantly on the properties of the adsorbent, including its surface area, porosity, and surface chemistry, as well as the physicochemical characteristics of the target pollutant (Dąbrowski 2001).

Activated carbon (AC) has long been regarded as the standard adsorbent material for adsorption-based water treatment due to its high surface area, porosity, and chemical stability. Derived from carbonaceous materials such as coal, wood, or agricultural waste, AC possesses a microporous structure that facilitates the entrapment of organic molecules (Perrich 2018). Its surface can be modified to enhance interactions with specific pollutants, making it highly versatile (Bhatnagar et al. 2013; Pego et al. 2019). AC has been extensively studied for the adsorption of phenolic compounds, with reported adsorption capacities for phenol exceeding 500 mg/g, depending on the preparation method and experimental conditions (Hwang et al. 2017). For BPA, AC’s hydrophobic nature and π-π interactions with the aromatic rings of BPA contribute to its effectiveness, with adsorption capacities exceeding 1000 mg/g (Javed et al. 2018). However, AC’s performance can be limited by factors such as pore blockage, slow kinetics, and challenges in regeneration, which increase operational costs. Additionally, the production of high-quality AC can be resource-intensive, prompting researchers to explore alternative adsorbents that offer comparable or superior performance at a lower cost or environmental footprint (Larasati et al. 2021).

Graphene oxide (GO), which is a derivative of graphene, has recently gained attention as a next-generation adsorbent due to its exceptional physicochemical properties. GO is a two-dimensional material characterized by a high surface area (theoretically up to 2600 m^2^/g) and abundant oxygen-based functional groups such as hydroxyl, epoxy, and carboxyl groups (Liu 2017; McAllister et al. 2007). These functional groups enable GO to interact with pollutants through hydrogen bonding, electrostatic interactions, and π-π stacking, making it particularly effective for adsorbing aromatic compounds like phenol and BPA (Kong et al. 2023; Nam et al. 2024a). GO’s advantages include its tunable surface chemistry and potential for functionalization, which allows for tailored interactions with specific pollutants (Englert et al. 2011; Wang et al. 2021). However, challenges such as high production costs, potential aggregation in aqueous solutions, and uncertainties regarding its environmental fate necessitate further investigation (Ali et al. 2023; Nam et al. 2024b). Over the years, many studies have explored activated carbon and graphene oxide synthesized from various sources and modified using diverse chemical and thermal treatments to enhance their adsorption performance for phenolic compounds. These modifications aimed to improve surface area and the availability of functional groups, all of which influence the adsorption capacity and mechanism. A comprehensive summary of these studies is presented in Table 1, where the adsorption capacities, experimental conditions, and surface areas are compared across a wide range of AC- and GO-based materials applied for phenol and bisphenol-A removal. This comparison highlights the significance of adsorbent origin, surface functionalization, and experimental parameters in optimizing removal efficiency.

**Table 1:** Efficiency of AC- and GO-based adsorbents in the removal of phenol and bisphenol-A from wastewater

Adsorbent	Adsorbent source	Surface modification	Experimental conditions (T °C, pH)	*Q* (mg/g)	Surface area (m^2^/g)	Reference
Activated carbon-based adsorbents-phenol adsorption studies						
AC-commercial	Shanxi Sincere Industrial Co, LTD, China	Iron impregnation	25, 7	20.00	-	Channa et al. (2019)
Granular AC-coal	Sichuan Nan-Ke Activated Carbon Co., Ltd. China	-	30, 7	169.91	700.57	Xie et al. (2020)
Powdered AC-coal		-	30, 7	176.58	542.33	
Powdered AC-coconut		-	30, 5	212.96	1025.02	
AC-commercial	Sinopharm Group Chemical Reagent Co., Ltd	-	20,7	69.95	1187.4	Sun et al. (2019)
		HNO_3_-oxidation (30 °C)	20,3	40.79	986.7	
		HNO_3_-oxidation (60 °C)	20,2	33.20	858.2	
		HNO_3_-oxidation (85 °C)	20,2	9.05	364.2	
AC-commercial	Sigma-Aldrich	-	28, -	500.00	900	Hwang et al. (2017)
AC-poplar	Korea	Thermal (700 °C) and chemical activation (KOH)		454.50	1908	
AC-pine				625.00	2711	
AC-oily sludge	Oil Refinery Sludge—storage tanks of Isfahan, Iran	Thermal (800 °C) and chemical activation (KOH)	25, 6	434.78	2263	Mojoudi et al. (2019)
Activated carbon-based adsorbents-bisphenol-A adsorption studies						
AC	Gilsonite was provided by Prince Energy	Thermal (900 °C) and chemical activation (KOH	-	1113.00	3851	Javed et al. (2018)
GP-AC	Sigma-Aldrich	Goethite iron-oxide impregnation	25, 7	1.00	717	Koduru et al. (2016)
AC	Oil palm fruit bunch waste Malaysia	Carbonization at (500 °C)	27, 7	41.98	86.62	Wirasnita et al. (2014)
AC	Coffee grounds waste	Thermal (500 °C) and chemical activation (ZnCl_2_)	26, 5.6	123.20	1039	Alves et al. (2019)
AC	Areca catechu husk wastes	Thermal (550 °C) and chemical activation (phosphoric acid)	-, 7	28.41	483.40	Yong et al. (2025)
Graphene oxide-based adsorbents-phenol adsorption studies						
GO	Merck, India	-	30, 7	10.23	312	Mukherjee et al. (2019)
GO	American Bay Carbon	-	-,7	25.00	-	Wang et al. (2018)
GO	United Nanotech Pvt. Ltd. (India)	-	35,7	1.82	-	Manna et al. (2019)
GO		GO-biochar	35,7	23.47	-	
GO-MB	Areej Al-Furat Co, Ltd in Iraq	Bentonite	25, 6	46.43	184.312	Ayoob et al. (2024)
GO	Research lab company	-	25, 2	48.54	30.12	Bibi et al. (2022)
GO-PAA		Polymer	25, 2	84.30	21.22	
Graphene oxide-based adsorbents-bisphenol-A adsorption studies						
GO	Beijing Boyu Technology Corporation of High-tech New Materials, China	-	25, 7	8.30	-	Wang et al. (2021)
Fe_2_O_3_-GO		Fe_2_O_3_		11.50		
rGO		-		9.60		
Magnetic rGO-1	Alfa Aesar Co	FeCl_3_ and FeSO_4_−7H_2_O	15, 6	92.98	310.04	Wang et al. (2016)
Magnetic rGO-2				71.66	145.74	
AB	Merck, India	-	, 7	65.87	51.28	Fuzil et al. (2022)
GO-AB		Alginate beads		83.33	7.19	
GO	Sigma-Aldrich	Cobalt ferrites	55, ~ 7	30.00	-	Fachina et al. (2022)
rGO-HD	Sigma-Aldrich	Hydrazine sulfate reduction	25, 6	150.20	130.2	Bahadi et al. (2024)
rGO-800		Thermal reduction (800 °C)		193.50	125.1	

The growing global demand for clean water, alongside increasingly strict regulations on pollutant discharge, emphasizes the urgency of developing efficient and sustainable remediation strategies. By comparing AC and GO, this research contributes to ongoing efforts in optimizing adsorbent selection and design, suggesting insights that could inform the development of next-generation treatment systems. Although numerous adsorbents have been explored for the removal of phenolic pollutants, many suffer from limited performance in saline environments and do not effectively address the challenges posed by structurally diverse compounds such as phenol and bisphenol-A. Furthermore, comparative studies evaluating both activated carbon and graphene oxide under identical conditions are scarce, particularly in relation to their interaction mechanisms and performance stability. This study addresses these gaps by systematically investigating the adsorption behavior of both AC and GO toward phenol and BPA, thereby offering insights into material selection for efficient treatment in complex water matrices. The rationale for selecting AC and GO lies in their complementary strengths and respective limitations. AC’s widespread application in existing water treatment applications makes it a valuable reference point and benchmark for evaluating new materials, while GO’s distinctive physicochemical properties position it as a potential alternative. This comparative study aims to assess whether novel nanomaterials like GO can surpass conventional adsorbents such as AC in practical applications. Key adsorption parameters, including adsorption capacity, isotherm behaviors, and the effects of pH, temperature, initial adsorbate concentration, and ionic strength, were investigated to provide a comprehensive performance profile. Additionally, insights into the molecular-level interactions between adsorbents and pollutants are expected to clarify strategies for improving selectivity, which are critical to achieving cost-effective and scalable water treatment solutions.

## Materials and methodologies

### Materials

Graphite flakes, natural (−10 mesh), 99.9%, were purchased from Alfa Aesar; sulfuric acid (H_2_SO_4_), 95%; potassium permanganate (KMnO_4_), 99%; phosphoric acid (H_3_PO_4_), 85%; hydrogen peroxide (H_2_O_2_), 30%; and activated carbon were obtained from “Research lab” company, while hydrochloric acid (HCl), 35–38%, was acquired from Scharlu.

### Synthesis of graphene oxide using modified Hammers’ methods

Graphene oxide was synthesized using a modified Hummers’ method. First, 96 mL of concentrated sulfuric acid (74.2 wt.%) was mixed with 24 mL of phosphoric acid (19.7 wt.%) in an ice bath. Then, 4 g of graphite powder and 12 g of potassium permanganate were slowly added to the acid mixture while stirring continuously. The suspension was heated in an oil bath at 95 ± 2 °C and kept under reflux for 30 min. Next, 100 mL of distilled water was gradually poured in, and stirring continued for another 30 min. To terminate the reaction, the mixture was cooled in an ice bath and treated with 300 mL of distilled water and 40 mL of hydrogen peroxide. The suspension was then diluted with a 20% hydrochloric acid solution and centrifuged at 5000 rpm for 30 min. The supernatant was removed, and the solid was washed repeatedly with distilled water until a neutral pH was reached. Finally, the graphene oxide was dried in an oven at 60 °C for 48 h (Alkhouzaam et al. 2020).

### AC and GO characterization

The commercial activated carbon and the synthesized graphene oxide were characterized through various physicochemical techniques. The morphological features of both materials were examined using scanning electron microscopy (SEM) (Nova™ Nano SEM 50 Series) and transmission electron microscopy (TEM), allowing visualization of their structural differences. Functional groups present on the surfaces of AC and GO were identified through Fourier transform infrared spectroscopy (FTIR), performed using a SHIMADZU-IRSpirit spectrometer within the 400–4000 cm^−1^ range. Elemental composition assessment was accomplished via energy-dispersive X-ray spectroscopy (EDX) to confirm the presence and distribution of constituent elements in the materials. Raman spectroscopy was conducted using a Thermo Fisher Scientific DXR Raman Microscope to investigate the vibrational characteristics and structural ordering of carbon-based materials. Additionally, the Brunauer–Emmett–Teller (BET) surface area analysis was performed with a Quantachrome Nova 3000 instrument to determine the specific surface areas of the two adsorbents, offering insights into their porosity and potential adsorption capacity.

### Batch adsorption studies

To conduct the adsorption experiments, a phenol stock was first prepared by dissolving 0.1 g of phenol and bisphenol-A in 1 L of distilled water, yielding a concentration of 100 mg/L. This solution served as the basis for preparing a series of diluted samples ranging from 0 to 10 mg/L, which were used to construct a calibration curve for phenol detection. Ultraviolet–visible spectroscope (SHIMADZU-UV-1280) was employed to quantify the phenol concentration in both standard and test samples, with absorbance measurements taken at wavelengths of 270 nm for phenol and 277 nm for bisphenol-A (Gholami-Bonabi et al. 2020). Batch adsorption experiments were performed in 100-mL glass bottles, each containing 40 mL of phenolic solution at a known concentration. A fixed amount of 0.04 g of either activated carbon or graphene oxide was introduced to each bottle, which was then agitated at a persistent speed of 165 rpm for 24 h using a rotary shaker (Brunswick Innova® 2100/2150). The influence of key experimental parameters, including pH (2, 4, 6, 8, 10, and 12), initial adsorbate concentration (1, 10, 20, 30, 40, 50, 60, 70, 80, and 100 mg/L), temperature (20, 30, and 40 °C), and ionic strength (0, 0.1, and 0.5 M), was systematically investigated. The pH of the solutions was adjusted using 0.1 M solutions of HCl or NaOH as needed. Upon completion of the adsorption period, samples were filtered through a 0.45-µm Ahlstrom syringe filter and analyzed for residual phenol and bisphenol-A content using UV–vis spectrophotometry at 270 nm and 277 nm, respectively. All experiments were performed in duplicate, and mean values were used for data interpretation. Standard error was computed for all replicated measurements. The percentage of phenolic compounds removal and the adsorption capacity of each material were subsequently calculated using the following equations:

1 $$\text{Removal }\left(\%\right)= \frac{{C}_{\mathrm{i}}-{C}_{\mathrm{f}}}{{C}_{\mathrm{i}}}\times 100$$

where *C*_i_ and *C*_f_ are the initial and final concentrations (mg/L) of phenol/bisphenol-A in the solution.

The adsorption capacity was calculated according to the following equation:

2 $$\text{Adsorption capacity }Q\mathrm{e}=\frac{\left({C}_{\mathrm{i}}-{C}_{\mathrm{f}}\right)\times V}{W}$$

where *C*_i_ and *C*_f_ are the initial and final concentrations (mg/L) of phenol/bisphenol-A in the solution, *V* is the volume of solution (mL), and *W* is the weight of adsorbent (mg).

### Adsorption isotherms

**Fig. 2:**
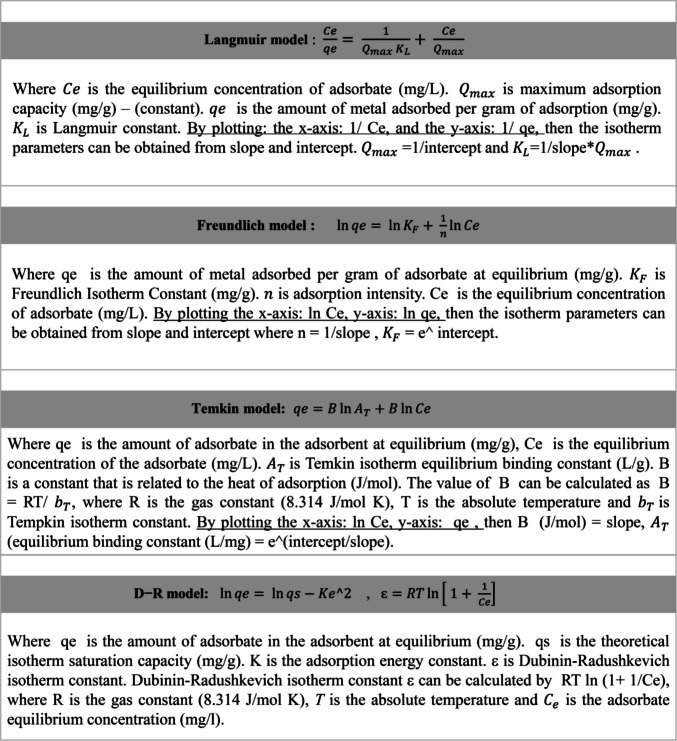
Adsorption isotherm models and their parameters

Adsorption isotherm studies were conducted to evaluate the equilibrium behavior of phenol uptake by the tested adsorbents under defined conditions of temperature and pH. These models describe the interaction between the adsorbate and adsorbent and provide insights into the adsorption mechanism and capacity at equilibrium. In the current investigation, a few widely recognized isotherm models were applied: Langmuir, Freundlich, Temkin, and Dubinin–Radushkevich (D–R), following the approaches (Kecili and Hussain 2018; Singh and Susan 2018) . The Langmuir model assumes monolayer adsorption onto a surface with a finite number of identical and energetically equivalent sites, without interactions between adsorbed molecules (Saleh 2022). In contrast, the Freundlich model is empirical and explains multilayer adsorption on heterogeneous surfaces, assuming a non-uniform distribution of heat of adsorption and interactions between adsorbed molecules (Krstić 2021). The Temkin model incorporates adsorbate–adsorbent interactions and assumes that the heat of adsorption decreases linearly with surface coverage, making it particularly relevant for systems where adsorbate–adsorbate interactions are non-negligible (Molina-Calderón et al. 2022). The Dubinin–Radushkevich (D–R) model, on the other hand, is often applied to distinguish between physical and chemical adsorption mechanisms; it assumes a Gaussian energy distribution onto a heterogeneous surface (Zhou et al. 2021). The experimental data were fitted to each model, and the corresponding parameters were determined to interpret the adsorption performance. Figure 2 summarizes the adsorption isotherm models and parameters.

### Thermodynamic studies

To evaluate the thermodynamic feasibility and mechanism of the adsorption process, the standard Gibbs free energy change (Δ*G*°), enthalpy change (Δ*H*°), and entropy change (Δ*S*°) were analyzed. A negative Δ*G*° indicates spontaneity and thermodynamic favorability, while a positive Δ*G*° suggests non-spontaneity. A positive Δ*H*° usually indicates an endothermic process, often linked to stronger adsorbate–adsorbent interactions, whereas a negative Δ*H*° implies exothermic behavior. A positive Δ*S*° reflects increased randomness at the solid–liquid interface, while a negative Δ*S*° suggests a more ordered system during adsorption. The above parameters were determined based on equilibrium constants derived from the Langmuir isotherm model. The Langmuir constant (*K*_L_) was obtained by nonlinear fitting of the isotherm data and has the unit L/mg. Since *K*_L_ is dimensional, it was first converted into a dimensionless standard equilibrium constant, *K*_L_°, before thermodynamic analysis. This transformation was performed according to the method proposed by Chen et al. (2021), by multiplying *K*_L_ by a reference standard concentration *C*° selected as 100 mg/L, which corresponds to the highest adsorbate concentration used in the experimental system:

3 $${K}_{L} ^\circ={K}_{L}\times C^\circ$$

The standard Gibbs free energy change was then calculated at each studied temperature (T, in Kelvin) using the following thermodynamic relation:

4 $$\Delta G^\circ=-RT\;ln\;K_L^\circ$$

*R* is the universal gas constant (8.314 J mol^−1^ K^−1^). The values of Δ*H*° and Δ*S*° were obtained from the slope and intercept, respectively, of the linear regression of ln *K*_L_° vs 1/T, based on the van’t Hoff equation (Chen et al. 2021):

5 $$\mathrm{ln}{K}_{L}=\frac{\Delta H^\circ }{R}\times \frac{1}{T}+\frac{\Delta S^\circ }{R}$$

### Statistical analysis

One-way analysis of variance (ANOVA) was applied to assess the effect of pH on adsorption performance. At the same time, two-way ANOVA was used to evaluate the effects of temperature, initial concentration, and adsorbent type in the batch experiments. All statistical analyses were performed using Microsoft Excel at a significance level of 0.05, and differences were considered statistically significant when *p* ≤ 0.05.

## Results and discussions

### Adsorbent characterization tests

#### Transmission electron microscopy (TEM)

**Fig. 3:**
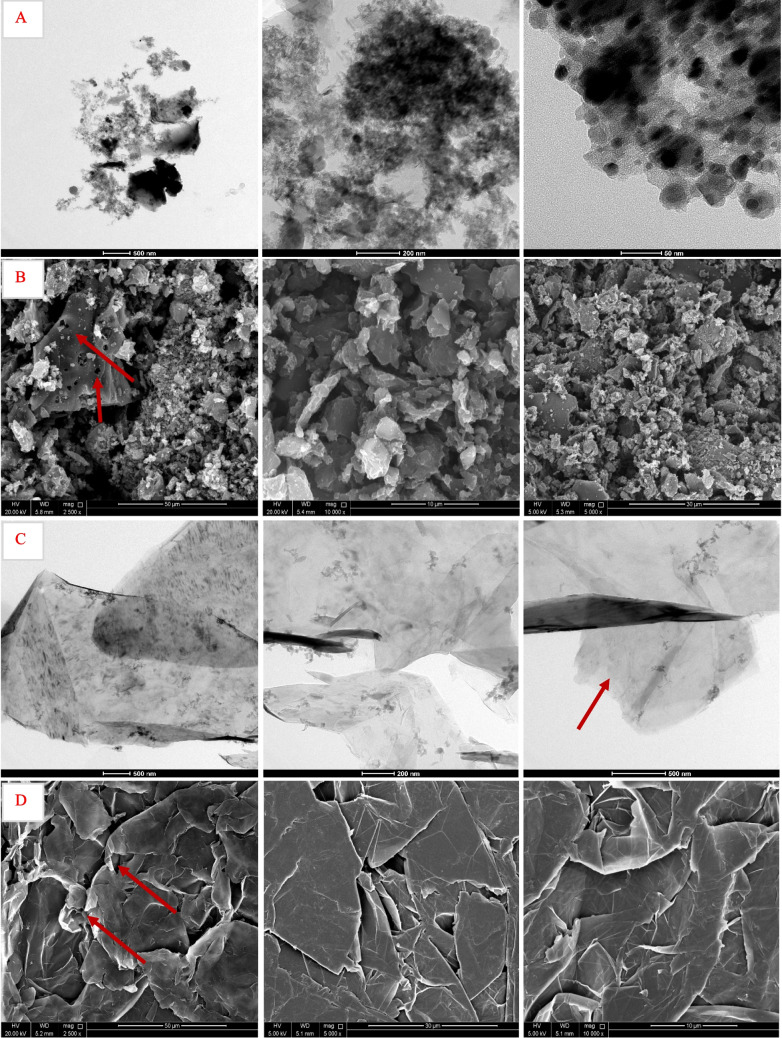
TEM and SEM images of AC (**A**, **B**) and GO (**C**, **D**)

The TEM images of activated carbon and graphene oxide shown in Fig. 3A and C show distinct structural features of each material. For AC, the TEM images display a highly amorphous structure indicative of a substantial surface area that is advantageous for adsorption. The images show irregular shapes and densities, highlighting the complex formation characteristic of AC. These irregularities in structure and the presence of micropores are essential for trapping various molecules, which makes AC highly effective in adsorption studies. In contrast, the TEM images of GO exhibit thin, more transparent sheets, often crumpled and overlapping, suggesting a flatter and few-layered structure. These ultra-thin layers contribute to GO’s surface area and flexibility, which are essential for various applications, including adsorption and filtration. The transparency and smoothness of the GO layers indicate a high degree of exfoliation and could also suggest the distribution of functional groups, which enhance its chemical reactivity (Abu-Nada et al. 2021).

#### Scanning electron microscopy (SEM)

The SEM images of activated carbon and graphene oxide further illustrate their unique morphologies, as shown in Fig. 3B and D. For AC, the SEM images show a rough and coarse texture with fragmented, rough particles, crevices, and pores, emphasizing its extensive surface irregularity. This roughness and porosity are critical for its effectiveness in adsorptive processes as they increase the material’s surface area, allowing for greater interaction with pollutants or other substances. Conversely, the SEM images of GO reveal a smooth, overlapping layered structure with visible ridges and folds. These crumpled sheets might also be an indication of a high level of oxidation achieved during the preparation of graphene oxide (Abu-Nada et al. 2021).

#### Energy-dispersive X-ray spectroscopy (EDX)

**Fig. 4:**
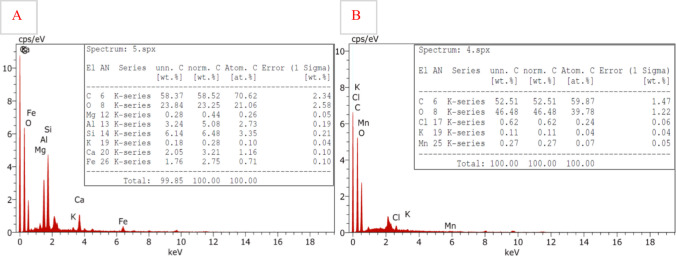
EDX elemental analysis of **A** activated carbon and **B** graphene oxide, showing their carbon and oxygen content along with trace elements

Energy-dispersive X-ray spectroscopy (EDX) analysis presents detailed information about the presence and concentration of elements within a sample. Figure 4A shows the EDX analysis of the commercial AC, which exhibited a carbon content of 58.37% by weight (70.62% by atomic percentage), confirming its carbon-rich nature. The presence of oxygen (23.84% by weight) indicates the presence of oxygen-containing functional groups, which can enhance the adsorption properties of AC. Additionally, several minor elements were detected, including aluminum (3.25%), silicon (2.15%), calcium (2.05%), iron (1.76%), and potassium (0.75%), suggesting that AC contains trace impurities or has been modified with additives to improve its structural integrity and adsorption performance. These additional elements can contribute to the overall porosity and surface chemistry of the material.

On the other hand, Fig. 4B illustrates the elemental composition of graphene oxide, which also displays a high carbon content of 52.51% by weight. The oxygen content was notably higher (46.48%) by weight, possibly indicating the introduction of oxygen-containing functional groups such as hydroxyl, epoxy, and carboxyl groups due to the oxidative treatment used during GO synthesis. These functional groups enhance GO’s hydrophilicity and reactivity, making it a promising material for applications in water treatment and as a precursor for further chemical modifications. Additionally, minor elements, including chlorine (0.62%), potassium (0.11%), and manganese (0.27%), were present in trace amounts. The detection of chlorine and manganese could be attributed to residuals from the oxidation process, particularly the use of potassium permanganate and hydrochloric acid during synthesis.

#### Fourier transform infrared spectroscopy (FTIR)

**Fig. 5:**
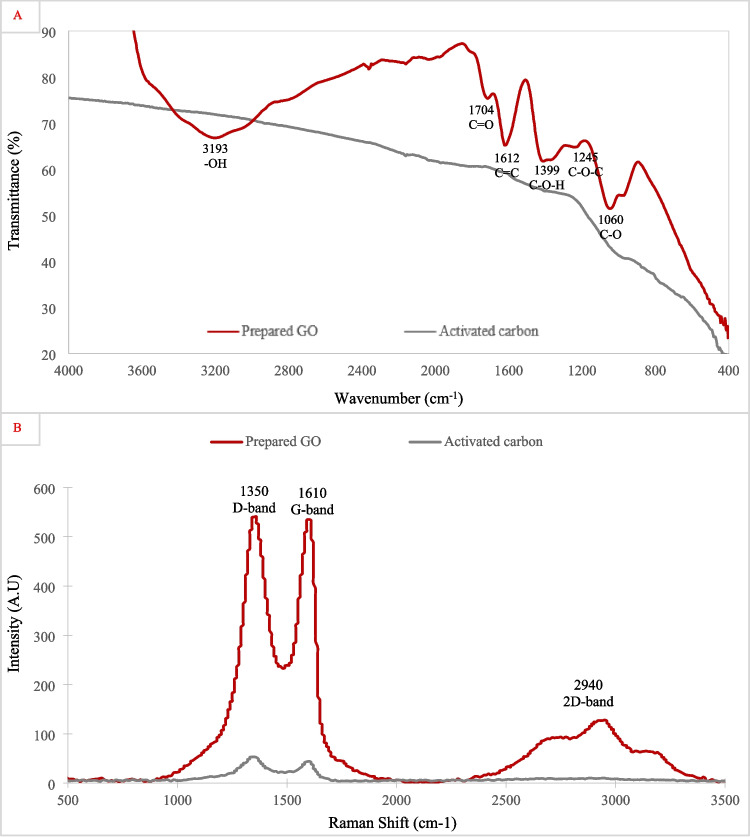
**A** FTIR spectra of AC and graphene oxide, highlighting the presence of oxygen functionalities. **B** Raman spectra of AC and GO showing the structural characteristics and disorder levels of the carbonaceous materials

FTIR spectroscopy was conducted to identify the functional groups present in the samples of activated carbon and graphene oxide. As shown in Fig. 5A, activated carbon displayed a smoother spectrum with fewer intense peaks, suggesting a lower concentration of oxygen-containing functional groups. The presence of residual surface functionalities is expected, as the EDX elemental analysis mentioned earlier indicated the presence of oxygen, but at a lower concentration compared to GO. This reduced intensity suggests a more carbonaceous structure. While activated carbon typically presents multiple peaks associated with surface oxygenated groups in the literature (Mojoudi et al. 2019), similar weakly defined FTIR spectra have also been reported (Nikravesh et al. 2023).

In contrast, GO exhibited multiple characteristic absorption bands, confirming the presence of oxygen-containing functional groups. The broad peak around 3200 cm^−1^ corresponds to O–H stretching vibrations, indicating hydroxyl groups from the oxidation process. The peaks near 1704 cm^−1^ can be attributed to the C = O stretching of carboxyl groups, while the bands around 1612 cm^−1^ correspond to C = C stretching from the graphitic backbone. Additionally, peaks near 1245 cm^−1^ and 1060 cm^−1^ are associated with C–O stretching vibrations of epoxy and alkoxy groups, respectively (Kanta et al. 2017).

#### Raman spectroscopy

Raman spectroscopy was employed to investigate the structural properties of activated carbon and graphene oxide, with the results illustrated in Fig. 5B. Both materials displayed the characteristic D and G bands, located at approximately 1350 cm^−1^ and 1610 cm^−1^, respectively. The D band is associated with structural defects and disorder in the carbon lattice, while the G band corresponds to the in-plane vibrations of sp^2^-hybridized carbon atoms (Vali et al. 2024). Activated carbon showed these bands with a relatively low intensity, with an intensity ratio of *I*_D_/*I*_G_ = 1.22, suggesting a more amorphous structure with defects, which is typical for well-carbonized materials. In contrast, GO exhibited a higher D/G intensity of the two peaks and an intensity ratio of *I*_D_/*I*_G_ = 1.01, also indicating disorder and structural defects introduced by oxidation. Additionally, GO showed peaks in the 2500–3000 cm^−1^ region, corresponding to overtone and combination modes, which further support the disruption of its sp^2^ carbon network. The appearance of these bands reflects enhanced vibrational coupling due to extensive structural disorder and confirms the presence of mixed vibrational modes resulting from oxidation-induced modifications or oxygen functionalities in the graphene oxide lattice (Krishnamoorthy et al. 2013).

#### Brunauer–Emmett–Teller (BET) surface area analysis

Table 2 provides data on multi-point BET specific surface area (SSA), single-point total pore volume, and single-point average pore radius of AC and GO. Activated carbon demonstrated a significantly higher specific surface area and total pore volume, with values of 535.951 m^2^/g and 0.6698 cm^3^/g, respectively. This indicates a greater potential for adsorption due to its extensive network of micropores and mesopores. Additionally, the smaller average pore radius of 25.0 Å suggests a higher proportion of micropores, which are particularly effective for adsorption due to their high surface area-to-volume ratio. Studies indicated that phenol adsorption mainly occurs via micropore filling driven by π–π dispersion interactions within pores smaller than twice the molecular diameter of phenol (Allahkarami et al. 2023). Given that the molecular size of phenol is approximately 0.43–0.57 nm and the pore size of AC is 2.5 nm, the adsorbent possesses sufficient pore dimensions to facilitate the diffusion of phenol into its internal structure.

**Table 2:** BET surface area, pore volume, and radius of the studied materials

Adsorbent	Multi-point BET SSA (m^2^/g)	Single-point total pore volume (cm^3^/g)	Single-point average pore radius, Å
Activated carbon	535.951	0.6698	25.0 Å (2.5nm)
Graphene oxide	18.057	0.08648	95.5 Å (9.55nm)

The structural characteristics of graphene oxide also suggested the potential for effective pore diffusion during the adsorption process. With a specific surface area of 18.057 m^2^/g, a total pore volume of 0.08648 cm^3^/g, and an average pore radius of 95.5 Å (9.55 nm), GO exhibited a mesoporous structure, as defined by IUPAC classifications (White et al. 2009). Given that the molecular size of phenol ranges between 0.43 and 0.57 nm, the pore dimensions of GO should be sufficiently large to allow both molecules to diffuse into its internal structure. The mesoporous nature of the material not only enabled surface adsorption but also likely facilitated intraparticle diffusion, allowing adsorbates to access internal adsorption sites. As summarized in Table 1, it was noted that AC in general had a higher surface area compared to GO-based adsorbents. This relatively low surface area could lead to a reduced number of available adsorption sites, potentially influencing the material’s adsorption performance and capacity in subsequent experiments.

Activated carbon adsorbents generally exhibited higher BET surface areas compared to graphene oxide-based materials in the studies mentioned in Table 1. In phenol adsorption studies, ACs showed surface areas ranging from approximately 364 to 2711 m^2^/g, with the highest values reported for pine- and oily sludge-derived ACs, both chemically and thermally activated (Hwang et al. 2017; Mojoudi et al. 2019). In contrast, GO-based adsorbents had lower surface areas, typically reaching 310 m^2^/g (Wang et al. 2016). Theoretically, graphene can have a surface area of around 2630 m^2^/g (Borand et al. 2021), but GO often falls short of this due to restacking and aggregation (Ali et al. 2023). AC can exceed this range in practice due to its irregularity, porosity, and successful activation methods. These studies show that while GO offers functional versatility, AC typically provides better surface area under experimental conditions, which often translates into higher adsorption capacities, especially when the pore size is suitable.

### Batch adsorption studies

#### Effect of pH

The pH of the experimental setup has a crucial role in determining the structural form, ionization state, and reactivity of organic pollutants, thereby significantly influencing their environmental behavior and interactions with adsorbents. Variations in pH can alter the speciation of these compounds, affecting their solubility, polarity, and potential for electrostatic or hydrophobic interactions (Li et al. 2022). For instance, phenol undergoes significant changes in speciation depending on the pH of the solution. At acidic to near-neutral pH, typically below pH 9.9, which is the pKa of phenol, it predominantly exists in its neutral molecular form. In this state, phenol is less hydrophilic and can engage more readily in hydrophobic interactions or π–π stacking with surfaces. However, as the pH increases above the pKa, phenol deprotonates to form phenolate anions. These anionic forms are more soluble in water due to increased polarity and are surrounded by a hydration shell, making them less likely to adsorb or interact with nonpolar or neutral surfaces. Moreover, the transition from a neutral molecule to a charged species at higher pH introduces repulsive forces when interacting with similarly charged environments, further decreasing the tendency for phase movement (Xie et al. 2020). Bisphenol-A also remains neutral and hydrophobic, with no ionization of the hydroxyl groups at acidic and neutral pH levels. However, in basic conditions, BPA’s phenolic group deprotonates, forming negatively charged ions, which increases its solubility and hydrophilicity (Li et al. 2022).

**Fig. 6:**
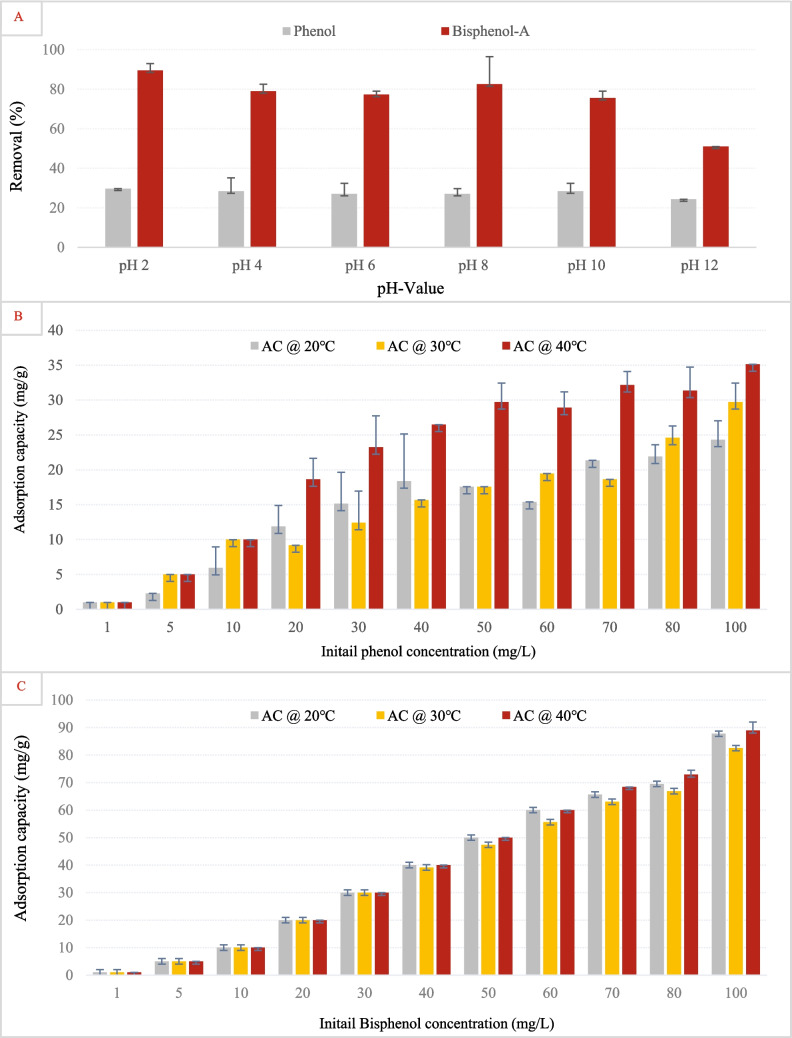
**A** Removal efficiency of phenol and bisphenol using graphene oxide at different pH values. Adsorption capacity of graphene oxide in removing **B** phenol and **C** bisphenol-A at varying initial concentrations and temperatures. Error bars represent standard error of the mean

Figure 6A illustrates the effect of pH on the removal efficiency of phenol and bisphenol-A using activated carbon. Across all pH values, BPA exhibited significantly higher removal than phenol. The removal of phenol remained consistently low, indicating that its adsorption onto AC was not strongly affected by pH variations. This aligns with the earlier discussion that phenol, although undergoing deprotonation above pH 9.9, retains limited adsorption potential due to increased polarity and hydration shell formation, which reduces its interaction with hydrophobic surfaces like AC. Bisphenol-A in general showed higher removal which could be due to its higher *K*_ow_ values and increased hydrophobicity compared to phenol (Ra et al. 2008). Even though BPA showed maximum removal at pH 2, with a gradual decline as pH increased, the overall difference or impact of pH was not highly significant. The enhanced removal at acidic pH likely reflects the predominance of neutral, hydrophobic BPA molecules that interact favorably with AC via π–π stacking and hydrophobic forces. As the pH increases and BPA deprotonates to form phenoxide anions, its increased solubility and negative charge likely introduce electrostatic repulsion with the partially negative AC surface, reducing adsorption efficiency. This behavior was also supported by many studies mentioned earlier in Table 1, where activated carbon used for phenol and BPA adsorption showed optimal performance at neutral pH ~ 7. High adsorption capacities were reported for coal- and coconut-based powdered ACs and KOH-activated AC from oily sludge and poplar, all tested around pH 7. This suggests that π–π interactions and hydrophobic effects were most effective when phenol and BPA mainly remained in their molecular forms.

**Fig. 7:**
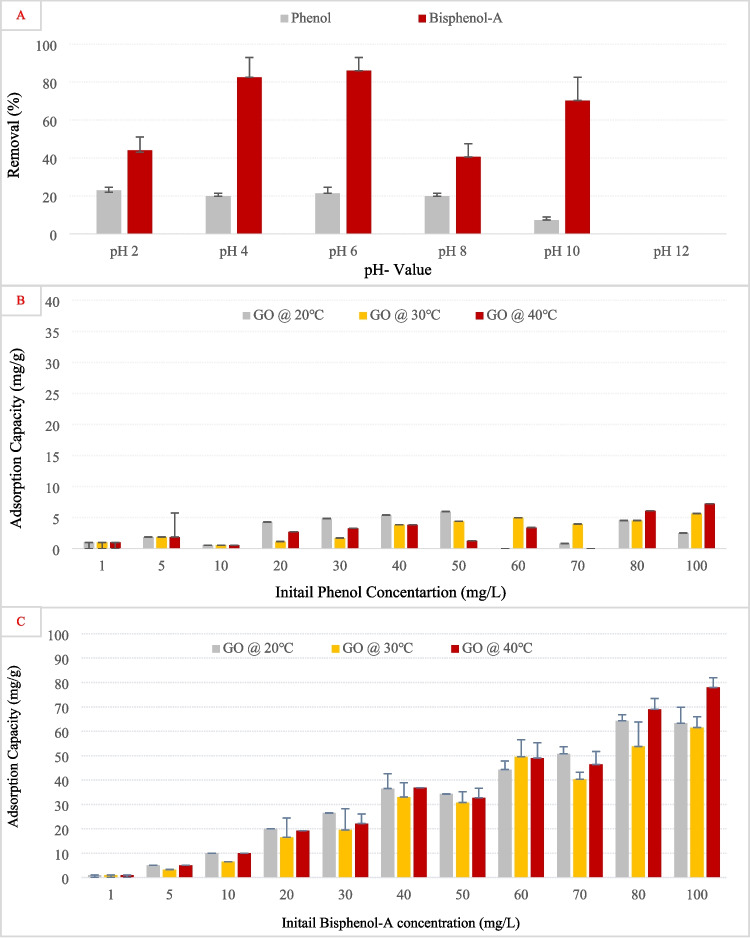
**A** Removal efficiency of phenol and bisphenol using activated carbon at different pH values. Adsorption capacity of activated carbon in removing **B** phenol and **C** bisphenol-A at varying initial concentrations and temperatures. Error bars represent standard error of the mean

Conversely, Fig. 7A presents the effect of pH on phenol and BPA removal using graphene oxide. As observed with AC, BPA consistently showed higher removal than phenol. Phenol adsorption remained low and largely unaffected by pH, supporting the idea that electrostatic interactions play a minor role in its interaction with GO. BPA removal, however, peaked at pH 6, suggesting an optimal balance between hydrophobic and electrostatic interactions. At this pH, BPA may remain neutral, facilitating stronger adsorption through π–π stacking and hydrogen bonding. The subsequent decline in removal at pH 10 and 12 is likely due to increased deprotonation of BPA, leading to repulsive interactions with the negatively charged GO surface (Li et al. 2022). These results highlight how pH-dependent speciation of phenolic compounds influences its interaction with adsorbents, consistent with its shift from neutral to anionic form under alkaline conditions.

#### Effects of initial pollutant concentration and temperature

Figure 6B presents the adsorption capacity of AC for phenol at different temperatures (20 °C, 30 °C, and 40 °C) as a function of the initial phenol concentration. A clear increase in adsorption capacity was observed with increasing phenol concentration. Mainly, the adsorption capacity was highest at 40 °C, indicating that the process might be endothermic, where higher temperatures enhance the mobility of phenol molecules by reducing their viscosity and promoting their kinetic interaction with AC (Darla et al. 2023). The lower adsorption at 20 °C suggests that diffusion limitations or weaker interactions could be restricting phenol uptake at lower temperatures. Figure 6C examines the adsorption capacity of AC for bisphenol-A under similar temperature conditions. A sharp increase in adsorption capacity was observed with increasing initial bisphenol-A concentration, reaching near saturation at the highest tested concentrations. Unlike phenol, bisphenol-A adsorption exhibited less temperature dependency, as the adsorption capacity remained relatively comparable across different temperatures. This behavior suggests that bisphenol-A adsorption onto AC is predominantly governed by hydrophobic interactions and π–π stacking, rather than temperature-driven diffusion effects. The high adsorption capacity of bisphenol-A compared to phenol further supports the hypothesis that bisphenol-A has a stronger affinity for AC, potentially due to its larger molecular structure, logK_ow_, and greater hydrophobicity, which enhances van der Waals forces and non-covalent interactions with the carbon surface.

Figure 7B presents the adsorption capacity of GO for phenol as a function of initial phenol concentration at different temperatures. Unlike activated carbon, the adsorption capacity of GO for phenol remained quite low across all concentrations and temperatures, indicating poor affinity between phenol and GO. This result suggests that phenol’s interaction with GO is weak, possibly due to a lack of strong π–π interactions or favorable surface functional groups. The slight variations in adsorption with temperature imply that temperature effects were not significant, which could mean that adsorption onto GO is not highly energy-dependent and is likely controlled by weak physisorption mechanisms. In a recent study, it was suggested that GO functionalization with a carboxyl group decreases its capacity for phenol adsorption (Ghahghaey et al. 2021). Figure 7C shows the adsorption capacity of GO for bisphenol-A at different temperatures and initial concentrations. A significant increase in adsorption capacity was observed with increasing bisphenol-A concentration, suggesting a strong interaction between GO and bisphenol-A. Compared to phenol, bisphenol-A demonstrated substantially higher adsorption onto GO, likely due to its larger molecular structure, which enhances van der Waals interactions, hydrophobic effects, and possible π–π interactions with the graphene structure. Specifically, the lying-down configuration of BPA on graphene oxide could provide the strongest adsorption due to the presence of dispersion forces and π–π coupling (Cortés-Arriagada et al. 2013). The adsorption capacity showed minimal temperature dependency, similar to the trend seen with activated carbon, suggesting that the process is primarily governed by non-covalent interactions rather than diffusion-controlled adsorption. The relatively high adsorption of bisphenol-A compared to phenol further reinforces the importance of molecular structure and hydrophobicity in determining adsorption efficiency on GO surfaces.

#### Effects of ionic strength

**Fig. 8:**
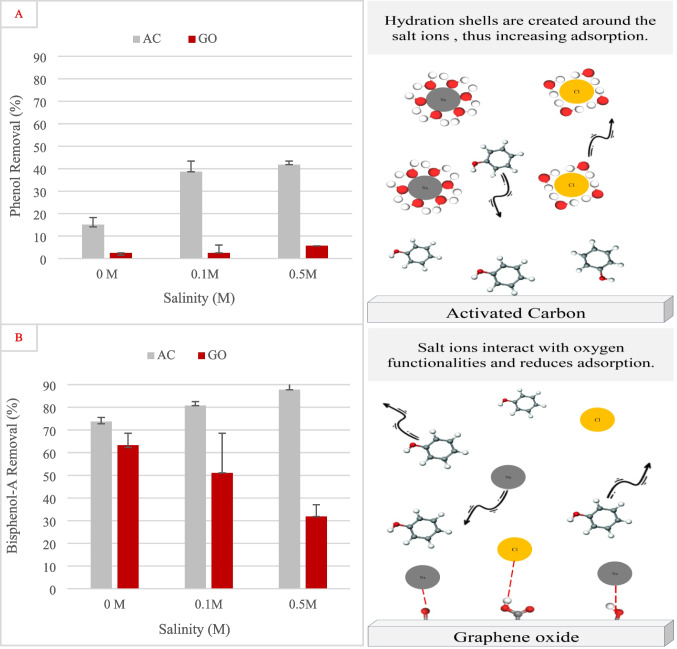
Effect of ionic strength on the removal of **A** phenol and **B** bisphenol-A using activated carbon and graphene oxide. Error bars represent standard error of the mean

The adsorption results revealed a clear difference in performance between activated carbon and graphene oxide for phenol and bisphenol-A removal under varying salinity conditions. For phenol, AC demonstrated significantly higher removal efficiency across all salinity levels, with removal increasing from approximately 15% at 0 M to over 40% at 0.5 M salinity. This tendency suggested that salinity enhanced phenol adsorption onto AC, possibly through the salting-out phenomenon (Zhang et al. 2019). At the molecular level, the addition of salt to phenol-containing water induces a salting-out effect, where salt ions such as Na⁺ and Cl^−^ preferentially interact with water molecules, reducing the availability of free water to solubilize phenol. This leads to decreased phenol solubility and promotes its migration toward the activated carbon surface, where it is more easily adsorbed through hydrophobic and π–π interactions (Lazo-Cannata et al. 2011). In contrast, GO exhibited minimal removal of phenol at all salinities, likely due to unfavorable interactions in the presence of salts. As illustrated in Fig. 8, when NaCl is added to the solution, Na⁺ and Cl^−^ ions can adsorb onto the functional groups present on the GO surface, such as carboxyl, hydroxyl, and epoxy groups. These sites are normally available for interacting with phenol through hydrogen bonding or electrostatic interactions. However, when occupied by salt ions, they become unavailable for phenol adsorption. As a result, the presence of NaCl occupies the GO surface, effectively blocking active sites and reducing the overall adsorption capacity for phenol. This mechanism leads to a salting-in effect in the sense that phenol remains in solution, not because of increased solubility, but due to competitive surface occupation by salt ions. A similar behavior was observed in a study where GO was used to alleviate salinity stress in tomato plants irrigated with a saline solution. It was found that GO adsorbed the salt ions, which in turn enhanced plant growth under stressful conditions (Yamada et al. 2024).

In the case of BPA, both AC and GO showed considerably higher removal efficiencies compared to phenol. AC maintained strong performance, with removal increasing slightly from about 75% to 85% as salinity increased. This behavior is consistent with the hydrophobic nature of BPA and its potential for π–π interactions with the AC surface. GO, however, displayed a decline in BPA removal, decreasing from approximately 65% at 0 M to around 30% at 0.5 M. This sensitivity to ionic strength may be attributed to the disruption of electrostatic and hydrogen bonding interactions, which are critical for GO’s adsorption mechanism. These findings highlighted that while AC performed consistently well for both phenolic compounds in saline environments, GO’s effectiveness reduced under such conditions, emphasizing the need for surface modification to enhance its performance in saline water treatment.

### Adsorption isotherm models and parameters

The adsorption isotherm data demonstrate the interactions between phenol or bisphenol-A and two adsorbents across four models at different temperatures, as summarized in Table 3. For phenol removal using AC, the Langmuir model showed high correlation coefficients (*R*^2^ = 0.95–0.99), indicating that monolayer adsorption on a homogeneous surface dominated the interaction. The maximum adsorption capacity *Q*_max_ reached 39.37 mg/g at 30 °C, suggesting favorable adsorption at moderate temperatures. The Langmuir constant, *K*_L_, is related to the binding affinity of adsorption and was highest at 40 °C (0.28 L/mg), indicating enhanced affinity between phenol and AC at the studied temperature (Al-Ghouti and Da’ana, 2020). The better fit of the Langmuir model for activated carbon indicated monolayer adsorption on a relatively homogeneous surface, which aligned with the high BET surface area found and the SEM analyses that previously showed porosity and a general distribution of active sites. The Freundlich model, while also fitting well (*R*^2^ = 0.95–0.99), revealed *n* values greater than 1 in almost all cases, indicating favorable physical adsorption on a heterogeneous surface (Al-Ghouti & Da’ana 2020). Based on the correlation coefficients, phenol adsorption onto AC followed both homogeneous and heterogeneous behavior, coinciding with several studies that mentioned that the behavior of phenol could be described by both models (Mojoudi et al. 2019). Temkin model results further supported the exothermic nature of the interaction, with heat of adsorption values *B* ranging between 5.79 and 10.46 J/mol and high linearity (*R*^2^ up to 0.97). In the D–R model, low *R*^2^ values (0.62), particularly at 30 °C, implied a poorer fit compared to the Langmuir and Freundlich models.

**Table 3:** Adsorption isotherm models and parameters for activated carbon and graphene oxide used for the removal of phenol and bisphenol-A

		Langmuir			Freundlich			
Phenol-AC	***T* (°C)**	***Q***_**max**_** (mg/g)**	***K***_**L**_** (L/mg)**	***R***^**2**^	***K***_**F**_** (mg/g) (L/g)**^n^	***n***	**1/*n***	***R***^**2**^
	20.00	29.49	0.06	0.97	6.62	3.34	0.30	0.95
	30.00	39.37	0.03	0.99	2.18	1.65	0.61	0.99
	40.00	34.84	0.28	0.95	17.64	6.33	0.16	0.97
		**Temkin**			**Dubinin–Radushkevich**			
	***T* (°C)**	***A***_**T**_** (dm**^**3**^**/mg)**	***B* (J/mol)**	***R***^**2**^	***Q***_**s**_** (mg/g)**	***K***	***R***^**2**^	
	20.00	0.84	5.79	0.97	4.00E + 08	−6.00E−05	0.76	
	30.00	0.19	10.46	0.95	6.60E + 09	−4.00E−04	0.62	
	40.00	3.95	5.90	0.95	9.30E + 12	−1.00E−04	0.95	
		**Langmuir**			**Freundlich**			
Phenol-GO	***T* (°C)**	***Q***_**max**_** (mg/g)**	***K***_**L**_**(L/mg)**	***R***^**2**^	***K***_**F**_** (mg/g) (L/g**^**) n**^	***n***	**1/*n***	***R***^**2**^
	20.00	5.88	1.24	0.36	3.32	10.87	0.09	0.22
	30.00	7.69	0.03	0.44	0.06	0.96	1.04	0.84
	40.00	5.46	5.23	0.54	2.94	6.71	0.15	0.13
		**Temkin**			**Dubinin–Radushkevich**			
	***T* (°C)**	***A***_**T**_** (dm**^**3**^**/mg)**	***B* (J/mol)**	***R***^**2**^	***Q***_**s**_** (mg/g)**	***K***	***R***^**2**^	
	20.00	0.84	5.79	0.22	4.00 × 10^8^	−6.00 × 10^−5^	0.09	
	30.00	0.07	3.22	0.81	1.81 × 10^2^	−5.00 × 10^−4^	0.77	
	40.00	22.56	0.77	0.12	3.65 × 10^2^	−2.00 × 10^−4^	0.18	
		**Langmuir**			**Freundlich**			
BPA-AC	***T* (°C)**	***Q***_**max**_** (mg/g)**	***K***_**L**_**(L/mg)**	***R***^**2**^	***K***_**F**_** (mg/g) (L/g)**^n^	***n***	**1/*n***	***R***^**2**^
	20.00	172.41	0.07	0.94	36.60	3.23	0.31	0.91
	30.00	82.65	0.49	0.93	37.34	3.83	0.26	0.94
	40.00	105.26	0.29	0.77	60.34	8.00	0.13	0.99
		**Temkin**			**Dubinin–Radushkevich**			
	***T* (°C)**	***A***_**T**_** (dm**^**3**^**/mg)**	***B* (J/mol)**	***R***^**2**^	***Q***_**s**_** (mg/g)**	***K***	***R***^**2**^	
	20.00	1.49	29.69	0.89	5.80 × 10^55^	−1.50 × 10^−3^	0.83	
	30.00	6.91	16.19	0.91	2.50 × 10^31^	−5.00 × 10^−5^	0.72	
	40.00	10.76	16.11	0.79	1.02 × 10^43^	−3.00 × 10^−4^	0.47	
		**Langmuir**			**Freundlich**			
BPA-GO	***T* (°C)**	***Q***_**max**_** (mg/g)**	***K***_**L**_**(L/mg)**	***R***^**2**^	***K***_**F**_** (mg/g) (L/g)**^**n**^	***n***	**1/*n***	***R***^**2**^
	20.00	70.92	0.17	0.92	21.33	3.57	0.28	0.61
	30.00	67.11	0.10	0.82	10.80	2.27	0.44	0.58
	40.00	63.29	0.56	0.94	23.57	3.84	0.26	0.17
		**Temkin**			**Dubinin–Radushkevich**			
	***T* (°C)**	***A***_**T**_** (dm**^**3**^**/mg)**	***B* (J/mol)**	***R***^**2**^	***Q***_**s**_** (mg/g)**	***K***	***R***^**2**^	
	20.00	3.40	12.38	0.57	6.07 × 10^22^	−7.00 × 10^−5^	0.46	
	30.00	0.67	16.64	0.75	3.73 × 10^20^	−2.00 × 10^−4^	0.12	
	40.00	6.48	11.08	0.37	1.32 × 10^21^	−9.00 × 10^−6^	0.26	

In contrast, phenol adsorption onto GO was poorly described by the Langmuir model, with very low *R*^2^ values (0.36–0.54). The adsorption capacity was significantly lower than with AC, peaking at only 7.69 mg/g. The Freundlich model showed a better fit only at 30 °C (*R*^2^ = 0.84). At other temperatures, *R*^2^ values were unsatisfactory, implying non-ideal phenol adsorption. The Temkin model also showed inconsistent and low *R*^2^ values. Similarly, the D–R model revealed low *Q*_s_ and very weak correlation (*R*^2^ ≤ 0.18) at 40 °C, suggesting poor suitability of this model for GO in phenol removal. Collectively, these results as well as the results of previous studies mentioned in Table 1 confirmed that AC outperformed GO in phenol adsorption, both in capacity and affinity.

For bisphenol-A removal, AC again showed better performance under the Langmuir model, with *Q*_max_ peaking at 172.41 mg/g at 20 °C and maintaining a high fit (*R*^2^ ≥ 0.77) across all temperatures. The highest affinity-based *K*_L_ was observed at 30 °C (0.49 L/mg). The Freundlich model presented *n* values between 3.23 and 8.00, indicating strongly favorable multilayer adsorption and relatively good correlation (*R*^2^ = 0.91–0.99) (Hamdaoui and Naffrechoux, 2007). According to a recent study, the heterogeneity factor (1/*n*) serves as an essential indicator of the adsorption mechanism. When the value of 1/*n* falls between 0 and 0.5, the adsorption process is considered favorable. In contrast, values between 0.5 and 1 suggest the presence of specific resistances or hindrances affecting adsorption efficiency. A 1/*n* value greater than 1 typically signifies that chemisorption is the dominant mechanism, which tends to make the adsorption of the adsorbate more difficult (Samadi et al. 2024). Based on Table 3, all 1/*n* values were within the favorable range except for the phenol adsorption using GO at 30 °C. The above results suggest that AC’s surface was highly efficient for BPA binding. The Temkin model again revealed a decreasing *B* value with increasing temperature and high *R*^2^ values (up to 0.91). The D–R model, despite indicating high *Q*_s_, showed limited fit (*R*^2^ < 0.83), making it less reliable for BPA adsorption behavior analysis.

For GO and BPA, the Langmuir model provided high values (63.29–70.92 mg/g) and relatively acceptable *R*^2^ values (0.82–0.94), particularly at 40 °C. This suggested better monolayer adsorption compared to phenol aligning with the sheet-like structures of GO shown earlier in Fig. 3C and D. Freundlich constants revealed favorable *n* values (> 2), although the model showed a poorer fit overall (*R*^2^ ≤ 0.61). To sum up, AC was consistently more efficient than GO in adsorbing both phenol and BPA, with better model conformity and higher adsorption capacities. Langmuir and Freundlich models were most appropriate for AC, revealing both monolayer and heterogeneous multilayer adsorption, respectively. For GO, adsorption was weaker and less model-compliant, particularly for phenol. The adsorption mechanisms mostly indicated physical interactions.

### Thermodynamic studies

The thermodynamic parameters, such as Gibbs free energy (∆*G*°), enthalpy (∆*H*°), and entropy (∆*S*°), can provide insight into the nature of phenol and bisphenol-A adsorption onto activated carbon and graphene oxide. For all adsorbent-adsorbate systems, the Gibbs free energy (∆*G*°) values were negative across the investigated temperature range (20–40 °C) as seen in Table 4, confirming the spontaneous nature of the adsorption processes. However, the magnitude of Δ*G*° varied considerably with both temperature and adsorbent type (Mojoudi et al. 2019). According to Bazan-Wozniak et al. (2022), the standard Gibbs free energy change (Δ*G*°) can also be used to differentiate between physisorption and chemisorption. Typically, Δ*G*° values ranging from − 20 to 0 kJ/mol are indicative of physical adsorption, while values between − 80 and − 400 kJ/mol are characteristic of chemical adsorption (Bazan-Wozniak et al. 2022). In the current study, all Δ*G*° values were within the range characteristic of physical adsorption, indicating that the adsorption process was primarily governed by physisorption.

**Table 4:** Thermodynamic parameters for the adsorption of phenol and bisphenol-A onto AC and GO at different temperatures

Sample	*T* (°C)	*T*(K)	1/*T* (K)	*K*_L_ °	ln (*K*_L_°)	∆*G*° (KJ)	∆*H*°	∆*S*°
Phenol-AC	20	293	0.003	6.00	1.79	−4.36	57,399.86	206.19
	30	303	0.003	2.74	1.01	−2.54		
	40	313	0.003	28.00	3.33	−8.67		
Phenol-GO	20	293	0.003	124.00	4.82	−11.74	51,122.79	202.03
	30	303	0.003	3.00	1.10	−2.77		
	40	313	0.003	523.00	6.26	−16.29		
BPA-AC	20	293	0.003	6.70	1.90	−4.63	57,183.69	214.25
	30	303	0.003	49.00	3.89	−9.80		
	40	313	0.003	29.23	3.38	−8.78		
BPA-GO	20	293	0.003	17.07	2.84	−6.91	44,629.55	172.85
	30	303	0.003	10.21	2.32	−5.85		
	40	313	0.003	56.43	4.03	−10.49		

Furthermore, the positive value of Δ*H*° reflects the thermal behavior of the adsorption process, where endothermic adsorption is indicated by a positive Δ*H*°, signifying heat absorption, while a negative Δ*H*° corresponds to an exothermic process, characterized by the release of heat during adsorption (Iftekhar et al. 2018). The positive Δ*H*° values observed for all systems indicate that the adsorption was endothermic. The increase in adsorption capacity with temperature supports the hypothesis of enhanced molecular mobility and diffusion into the porous structure of the adsorbents at higher temperatures.

In addition, positive entropy change (Δ*S*°) values reflect a strong interaction between the solid and liquid phases (Al Ashik et al. 2023), suggesting an increase in randomness or disorder in the distribution of pollutant molecules at the solid–liquid interface (Rincón-Silva et al. 2015). The entropy changes Δ*S*° were also positive for all systems, indicating an increase in randomness at the solid–solution interface during adsorption.

### Statistical analysis

The effect of pH on the removal efficiency of bisphenol-A and phenol by activated carbon and graphene oxide was evaluated using one-way ANOVA in Microsoft Excel 2018, and the results are summarized in Table 5. For phenol removal, the effect of pH was not significant for AC. However, it was significant for GO, suggesting that pH played a greater role in modulating phenol adsorption by GO than by AC. For BPA removal, the effect of pH was statistically significant for both AC and GO, indicating that pH strongly influenced adsorption performance.

**Table 5:** One-way and two-way ANOVA results showing the effect of experimental conditions on the removal efficiency of bisphenol-A and phenol using activated carbon and graphene oxide

Adsorbate	Parameter	Adsorbent	*F*-value	*p*-value	Significance (*α* = 0.05)
One-way ANOVA for the effect of pH					
Phenol	pH	AC	0.62	6.22 × 10^−1^	Not significant
		GO	5.39	1.30 × 10^−3^	Significant
BPA		AC	13.13	2.00 × 10^−4^	Significant
		GO	21.21	1 × 10^−5^	Significant
Two-way ANOVA for the effect of temperature and concentration					
Phenol	Concentration	AC	8.10	9.60 × 10^−5^	Significant
	Temperature		10.85	8.10 × 10^−4^	Significant
	Concentration	GO	7.54	1.50 × 10^−4^	Significant
	Temperature		4.36	2.90 × 10^−2^	Significant
BPA	Concentration	AC	88.73	7.10 × 10^−9^	Significant
	Temperature		7.73	3.77 × 10^−3^	Significant
	Concentration	GO	4.87	2.11 × 10^−3^	Significant
	Temperature		10.98	7.60 × 10^−4^	Significant
Two-way ANOVA for the effect of adsorbent type and concentration					
Phenol	Concentration	AC and GO	2.23	1.24 × 10^−1^	Not significant
	Adsorbent		28.18	4.88 × 10^−4^	Significant
BPA	Concentration		1.88	1.80 × 10^−1^	Not significant
	Adsorbent		69.87	1.56 × 10^−5^	Significant

Two-way ANOVA was also performed to examine the influence of initial adsorbate concentration and temperature on removal efficiency. For all adsorbent and adsorbate combinations, temperature exhibited statistically significant effects (*p* < 0.05) on removal efficiency. This indicated that variations in this factor led to measurable changes in the adsorption performance, regardless of the initial concentration or the target compound. On the other hand, at a temperature of 30 °C, the type of adsorbent also exhibited a statistically significant effect on removal efficiency, with *p* < 0.05. This indicated that the choice of adsorbent material played a crucial role in determining adsorption performance under these conditions. The observed differences suggested that variations in surface chemistry and specific interaction mechanisms between the adsorbent and the target molecules can influence pollutant removal. Therefore, it is essential to optimize the operational parameters mentioned above to achieve maximum removal efficiencies in adsorption-based treatment systems.

### Adsorption mechanisms

**Fig. 9:**
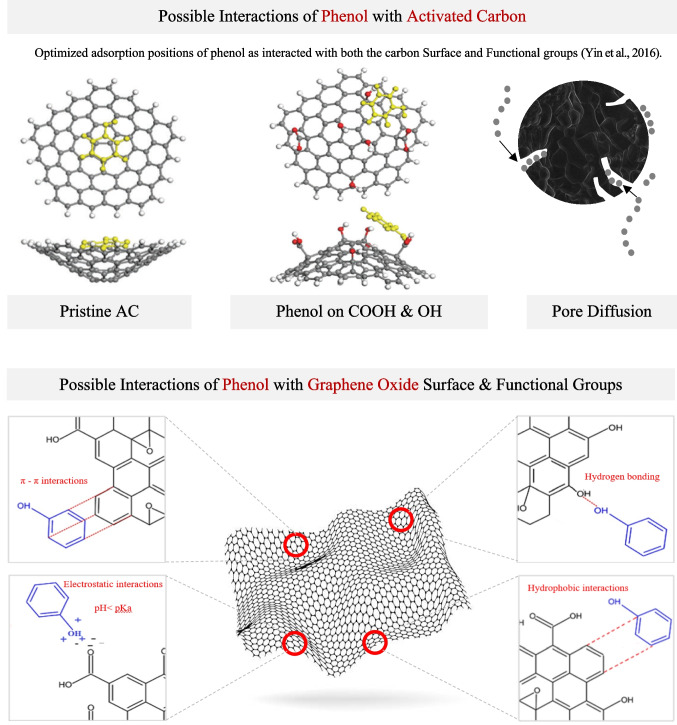
Possible interactions of phenol with activated carbon and graphene oxide through various adsorption mechanisms

Figure 9 presents a comparative illustration of the potential adsorption mechanisms governing the interaction of phenol with activated carbon and graphene oxide. For activated carbon, the adsorption process primarily involves π–π interactions between the delocalized electrons of phenol’s aromatic ring and the conjugated π-electron system on the graphitic planes of AC. When AC is functionalized with surface groups such as carboxyl (–COOH) and hydroxyl (–OH), phenol molecules can form hydrogen bonds. The porous structure of AC also facilitates phenol transport and entrapment through pore diffusion, especially within micro- and mesoporous regions, enhancing its sorption capacity. In the current study, AC showed superior adsorption performance for phenol compared to GO, even though no clear peaks corresponding to surface functional groups were observed in its FTIR spectrum. This enhanced adsorption was likely driven by strong π–π interactions between the aromatic ring of phenol and the extended conjugated carbon domains of AC. Furthermore, the high surface area and well-developed microporosity of AC may have contributed to effective pore diffusion and physical entrapment of phenol molecules, compensating for the lack of chemical functional groups (Allahkarami et al. 2023).

In general, graphene oxide has a heterogeneous surface enriched with oxygenated functionalities, including epoxy, hydroxyl, and carboxyl groups, which could introduce multiple binding sites for phenol (Ghahghaey et al. 2021; Yu et al. 2020). These functionalities allow for a broader range of interactions: π–π stacking similar to AC, but also strong hydrogen bonding and electrostatic interactions. In acidic or neutral conditions, protonated phenol may interact with negatively charged GO sites, while at higher pH, deprotonated phenolate ions can be repelled, as noted earlier. Moreover, hydrophobic interactions between the aromatic regions of phenol and the graphitic domains of GO further support adsorption. However, in this study, the GO, although rich in oxygenated functionalities, exhibited lower adsorption, possibly due to limited surface area and the disruption of the π-conjugated network by oxygen functional groups. The presence of surface oxygen groups, such as carboxyl groups in GO, also makes it more hydrophilic, which can lead to competitive adsorption with water molecules (Zhao et al. 2023), thus potentially reducing phenol removal.

### Limitations, implications, and future research directions

This study contributes to the growing body of research on the removal of phenol and bisphenol-A using carbon-based and graphene oxide-based adsorbents, offering novel insights into their comparative performance under saline conditions. Activated carbon demonstrated superior adsorption capacity compared to graphene oxide, with values that fall within or exceed many previously reported in the literature, as summarized in Table 1. For instance, powdered ACs derived from coal and coconut sources showed adsorption capacities between 169.91 and 212.96 mg/g at neutral or slightly acidic pH (Xie et al. 2020), while highly engineered ACs prepared from oily sludge or pine reached up to 625 mg/g (Mojoudi et al. 2019). In contrast, most unmodified GO adsorbents exhibited lower adsorption capacities, typically ranging from 1.82 to 25 mg/g (Mukherjee et al. 2019; Manna et al. 2019). However, certain surface-modified forms, such as GO-PAA and rGO-800, achieved higher capacities of 84.30 and 193.50 mg/g, respectively.

These comparisons underscore the consistent and high performance of AC without the need for extensive surface modification, highlighting its economic and operational advantages. Unlike many earlier studies conducted under freshwater or deionized conditions, the present work also addresses saline environments, a context highly relevant for industrial and coastal wastewater treatment but still underrepresented in the literature. This added dimension enhances the real-world applicability of the findings and strengthens the case for selecting AC in saline water treatment scenarios.

The performance of AC in this study suggests strong practical potential for its implementation in real wastewater treatment systems, particularly for industrial effluents, petrochemical discharges, and marine or brackish water streams where phenolic pollutants are prevalent. Its high adsorption capacity, structural robustness, and relatively low cost make it a promising candidate for integration into scalable treatment units or filtration systems. While GO exhibited lower adsorption performance under the studied conditions, its tunable surface functionality could still offer advantages in tailored applications or when used as part of composite materials targeting specific contaminants.

Despite the favorable results, certain limitations should be acknowledged. Further investigation is needed to assess the regeneration efficiency of AC, its adsorption stability across multiple reuse cycles, and its effectiveness in the presence of complex contaminant mixtures commonly found in real wastewater. Additionally, although the study demonstrated strong batch-scale performance, scaling up to pilot-scale systems is essential to evaluate operational feasibility, long-term performance, and cost-effectiveness under continuous flow conditions. Future work should also consider the development of mixed or composite adsorbents that combine the advantages of AC with the functional versatility of graphene-based materials. Finally, detailed kinetic studies under dynamic conditions are recommended to deepen understanding of adsorption rates and mechanisms, which are critical for the design and optimization of real-time treatment processes.

## Conclusions

This study presented a comparative evaluation of activated carbon and graphene oxide for the adsorption of phenol and bisphenol-A from aqueous solutions under varying physicochemical conditions. The results demonstrated that AC consistently outperformed GO in adsorption capacity, efficiency, and model conformity, particularly under conditions of elevated salinity and temperature. The highest adsorption capacities observed were about 39.37 mg/g for phenol and 172.41 mg/g for BPA on activated carbon. Under optimal conditions, graphene oxide removed approximately 7.69 mg/g of phenol and 70.92 mg/g of BPA. For phenol, AC showed favorable adsorption due to its high surface area and pore volume. At the same time, GO exhibited limited interaction, likely due to weaker π–π interactions and competition with water molecules for surface sites. In the case of BPA, both adsorbents achieved higher removal efficiencies than phenol, with AC again showing superior performance even under saline conditions. Isotherm and thermodynamic analyses confirmed that the adsorption processes were spontaneous and primarily of physical nature, with AC aligning well with both the Langmuir and the Freundlich models, as indicated by the higher correlation coefficients. Although GO exhibited moderate performance for BPA under specific conditions, its adsorption behavior was more variable and less predictable.

## Data Availability

The manuscript has associated data included in the body of the article.
